# Bunyavirus requirement for endosomal K^+^ reveals new roles of cellular ion channels during infection

**DOI:** 10.1371/journal.ppat.1006845

**Published:** 2018-01-19

**Authors:** Samantha Hover, Becky Foster, Juan Fontana, Alain Kohl, Steve A. N. Goldstein, John N. Barr, Jamel Mankouri

**Affiliations:** 1 School of Molecular and Cellular Biology, University of Leeds, Leeds, United Kingdom; 2 Astbury Centre for Structural Molecular Biology, University of Leeds, Leeds, United Kingdom; 3 MRC-University of Glasgow Centre for Virus Research, Glasgow, United Kingdom; 4 Loyola University Chicago Stritch School of Medicine, Maywood, Illinois, United States of America; Harvard Medical School, UNITED STATES

## Abstract

In order to multiply and cause disease a virus must transport its genome from outside the cell into the cytosol, most commonly achieved through the endocytic network. Endosomes transport virus particles to specific cellular destinations and viruses exploit the changing environment of maturing endocytic vesicles as triggers to mediate genome release. Previously we demonstrated that several bunyaviruses, which comprise the largest family of negative sense RNA viruses, require the activity of cellular potassium (K^+^) channels to cause productive infection. Specifically, we demonstrated a surprising role for K^+^ channels during virus endosomal trafficking. In this study, we have used the prototype bunyavirus, Bunyamwera virus (BUNV), as a tool to understand why K^+^ channels are required for progression of these viruses through the endocytic network. We report three major findings: First, the production of a dual fluorescently labelled bunyavirus to visualize virus trafficking in live cells. Second, we show that BUNV traffics through endosomes containing high [K^+^] and that these K^+^ ions influence the infectivity of virions. Third, we show that K^+^ channel inhibition can alter the distribution of K^+^ across the endosomal system and arrest virus trafficking in endosomes. These data suggest high endosomal [K^+^] is a critical cue that is required for virus infection, and is controlled by cellular K^+^ channels resident within the endosome network. This highlights cellular K^+^ channels as druggable targets to impede virus entry, infection and disease.

## Author summary

To penetrate cells and cause disease most viruses traverse the cellular endocytic network and exploit its environment to trigger the release of their genetic material into the cytosol. Endosomal pH, virus-receptor interactions, proteolytic cleavage, and lipid composition of membranes have all been previously reported to act as endosomal triggers. Here, we reveal the identity of an additional critical endosomal trigger for virus entry, namely a requirement for exposure to specific K^+^ ion concentrations, which we demonstrate is regulated by cellular K^+^ channels. We show that by blocking K^+^ channels, we can disrupt the K^+^ concentration across the endosomal system, impeding virus trafficking. Here, we reveal cellular K^+^ channels as a new druggable target to inhibit virus entry, and thus disease.

## Introduction

The *Bunyaviridae* family contains over 350 named members that are separated into 5 genera namely *Orthobunyavirus*, *Hantavirus*, *Nairovirus*, *Phlebovirus* and *Tospovirus* [1,2]. Together, these viruses infect humans as well as an array of animals, insects and plants, in which select members cause severe or fatal disease [2,3].

All bunyaviruses are enveloped and possess a negative sense single-stranded RNA (ssRNA) genome, which is trisegmented and encapsidated by the viral nucleoprotein to form ribonucleoproteins (RNPs) [3]. Bunyaviruses are generally spherical particles with a diameter between 80–140 nm containing spike-like projections of 5–10 nm composed of two transmembrane glycopolypeptides, Gn and Gc. Key stages of the virus lifecycle are shared across bunyavirus family members in mammalian hosts, including virion movement through the endocytic pathway during virus entry [4], and fusion of the virus envelope with endosomes permitting release of RNPs into the cytosol [5–8]. Bunyaviruses accomplish this by membrane fusion, mediated by Gc, a Type II fusion protein. Acidification alone is not always sufficient to trigger the fusion of specific bunyaviruses, whilst for other RNP containing viruses, the low endosomal pH is required for virus fusion, but alone is not sufficient [9]. Virus-receptor interactions, proteolytic cleavage of viral envelope glycoproteins and the lipid composition of endosomal membranes have all been postulated as additional factors [10]. However, the receptors, pathways and cellular factors required during bunyavirus endosomal progression remain largely unidentified and poorly characterized.

A recent report described how the productive infection of another (–) ssRNA virus, influenza A virus (IAV) is dependent on endosomal pH and potassium concentration ([K^+^]) during endosomal trafficking [11]. A K^+^ gradient from the intracellular to extracellular environment exists in all cells (~5 mM extracellular, ~150 mM intracellular) and [K^+^] is known to increase with passage along the endocytic pathway [10]. It was shown that both a drop in pH and gradual change in the overall ionic milieu in maturing endocytic vesicles were needed for virus infection. IAV infection and endosomal escape was far less efficient in the absence of high [K^+^] [11]. The cellular regulation of this ionic balance was not investigated.

Using the prototypic orthobunyavirus Bunyamwera virus (BUNV), we recently identified a critical role for cellular K^+^ channels during the BUNV infectious cycle [12]. We also identified a similar K^+^ channel dependence for other bunyaviruses namely Schmallenberg virus (genus *Orthobunyavirus*) and Hazara virus (genus *Nairovirus*), the latter is a close relative of the highly pathogenic Crimean Congo Hemorrhagic Fever Virus CCHFV [13]. We demonstrated that K^+^ channel blocking compounds could impede these bunyaviruses and showed that the stage of the lifecycle at which K^+^ channel activity is needed is after the time when the virus has entered the cell, but prior to mRNA transcription or RNA replication [12]. This implicated the functionality of K^+^ channels during BUNV movement through the endocytic machinery.

Here, we used BUNV as a tool to decipher why K^+^ channels are required for viral progression through the endosomal system. To achieve this, we generated dual-labelled BUNV that allows single virions to be tracked in cells, in the presence of K^+^ channel modulation. Our data indicates that BUNV traffics through endosomes containing high [K^+^] and that these K^+^ ions influence the infectivity of virions. We further show that K^+^ channel inhibition can alter the distribution of K^+^ across the endosomal system and arrest virus trafficking in endosomes. These studies reveal why K^+^ channels facilitate bunyavirus infection, which may represent a mechanism conserved across multiple virus families, highlighting the potential of K^+^ channels as druggable anti-viral targets.

## Results

### K^+^ ions influence virus infectivity

**Fig 1:**
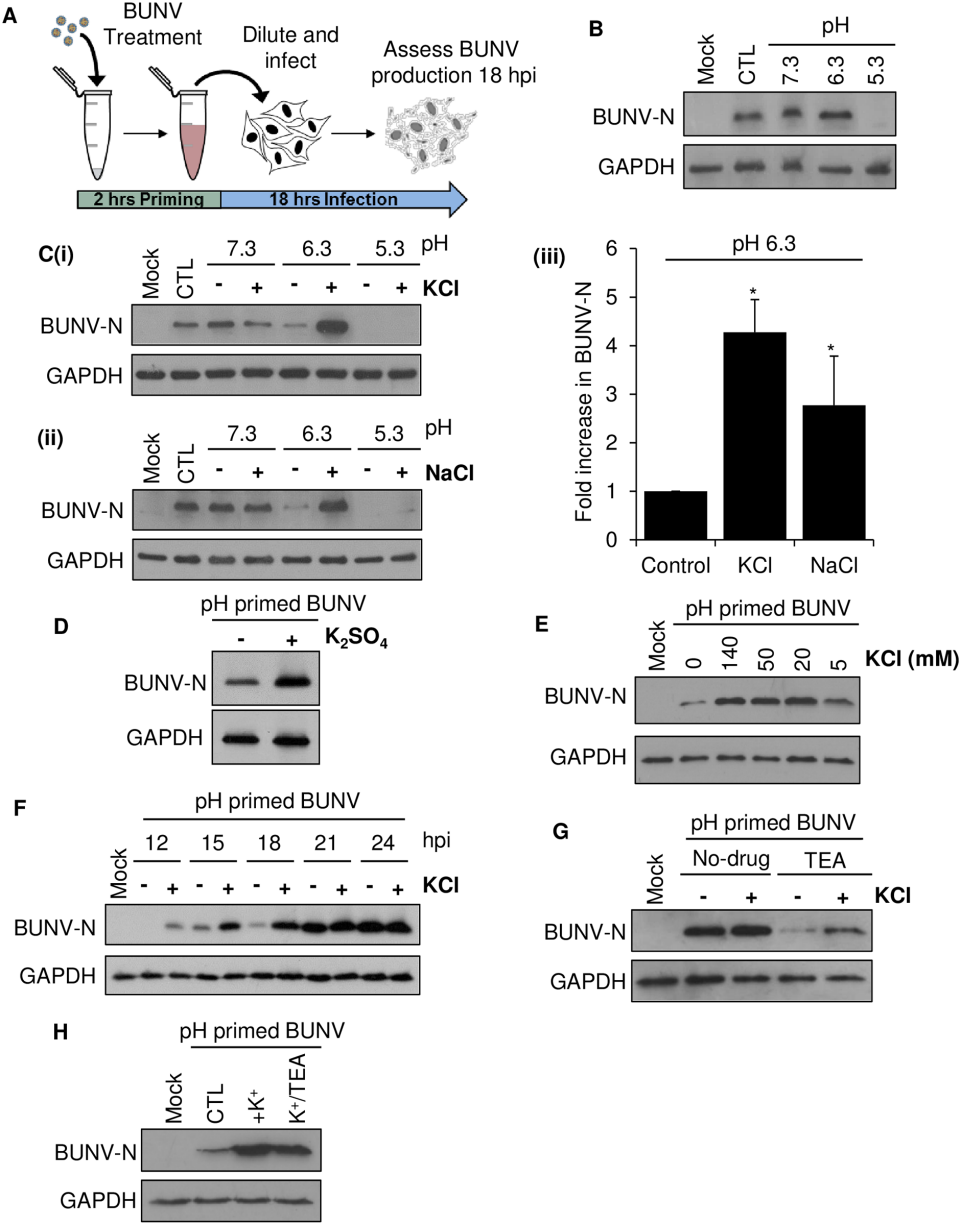
K^+^ ions at pH 6.3 expedite BUNV infection. **(A)** Schematic protocol of BUNV priming at 37°C with buffers of varying pH and salt concentrations for 2 hrs. Buffers were subsequently diluted out with media and A549 cells were infected with treated virions for 18 hrs prior to cell lysis. Western blot analysis was performed on cell lysates using BUNV-N protein as a marker of BUNV infection and GAPDH as a loading control. **(B)** Cells were infected with BUNV that had been pre-treated with buffers at pH 7.3, 6.3 or 5.3 (no salt). Cells were lysed and analysed by western blot, as in ***A*** (n = 3). **(C)** BUNV virions were treated with pH 7.3, 6.3 and 5.3 buffers with and without 140 mM **(i)** KCl or **(ii)** NaCl. Cells were infected with treated BUNV diluted in media and lysates were analysed by western blot, as in ***A*** (n = 3). **(iii)** Levels of BUNV-N at pH 6.3 under the indicated conditions were quantified by densitometry (*n* = 3). All error bars indicate mean ± SEM. *Significant difference from control (*P* < 0.05). **(D)** As with ***C***, using a pH 6.3 buffer with and without 140 mM K_2_SO_4_ (n = 3). **(E)** As with ***C***, using a pH 6.3–6.7 buffer with and without the indicated concentrations of KCl (n = 3). **(F)** A549 cells were infected with WT or pH 6.3/high KCl treated BUNV for 1 hr and non-internalized virions removed. Infection was allowed to proceed and cells lysed at 3 hr intervals from 12–24 hpi. Western blot analysis (as in ***A***) was carried out to compare WT and pH 6.3/High KCl treated BUNV-N production (n = 3). **(G)** BUNV virions were treated with a pH 6.3 buffer with and without 140 mM KCl, as in ***A***. A549 cells were treated 30 min prior to infection with 5 mM TEA and cells infected with the treated virions (with and without KCl) in the presence or absence of TEA throughout infection. Cells were lysed 18 hpi and BUNV-N production assessed as in ***A*** (n = 3). **(H)** BUNV virions were treated at pH 6.3 in the presence of 140 mM KCl with or without TEA 10 mM in the priming buffer. Buffers and drug were subsequently diluted out with media and A549 cells infected for 18hrs and BUNV-N levels assessed as in **A** (n = 3).

Given that BUNV requires K^+^ channels to progress through endosomes [12], we investigated whether endosomal ionic conditions were important during the BUNV lifecycle. We first addressed whether endosomal pH alone influenced the entry stages of BUNV infection by exposing purified virions *in vitro* to a range of buffers that mimic the pH of early endosomes (EEs) down to late endosome (LEs) and lysosomes (~6.3 to ~5.3). Following treatment, buffers were diluted with media and cells were infected with the treated viruses (MOI = 0.1) for 18 hrs; any effects on BUNV infection processes are therefore elicited by direct effects on the virions and not through changing the cellular environment (depicted in Fig 1A). This technique is referred to as acid-priming whereby mimicking the endosomal cues for virus penetration into the cytosol leads to an enhancement of virus infection [11]. We observed that pre-treatment of BUNV virions at pH 7.3 or pH 6.3 did not alter the timecourse or production of BUNV-N protein (a well characterised marker of BUNV infection), suggesting that exposure to pH in this range does not influence the infectivity of BUNV virions (Fig 1B). In contrast, incubation at pH 5.3 (that would mimic the pH of lysosomes) was strongly inhibitory and led to a complete loss of BUNV-N production, (Fig 1B, compare lanes 1–5) suggesting that pre-incubation at pH 5.3 blocked the infection process entirely (Fig 1B). We next addressed whether the predicted increase in endosomal [K^+^] influenced BUNV virions *in vitro*. When BUNV was pre-exposed to high [K^+^] at pH 7.3, minimal effects on virus infection were observed (Fig 1C(i)). However at pH 6.3 in high [K^+^], an increase in BUNV infection was identified, compared to the low [K^+^] buffer (Fig 1C(i), lanes 2–6). Na^+^ was also able to enhance the infectivity of BUNV virions at pH 6.3 though to a lesser extent than K^+^ (Fig 1C(i)–1C(iii)). Since both K^+^ and Na^+^ buffers contained Cl^-^ salts we ruled out its involvement as a similar enhancement occurred in K_2_SO_4_ based [K^+^] buffers (Fig 1D). At pH 5.3, viruses were again non-viable regardless of the presence of high [K^+^] or [Na^+^]. The rise in infection at pH 6.3 occurred in K^+^ buffers ranging from 20-140mM (Fig 1E). This was of interest as the luminal [K^+^] is proposed to increase during endosome maturation, reaching ≥ 50 mM in LEs [11,14]. Thus the observed effects of K^+^ occur at physiologically relevant endosomal [K^+^].

We next investigated the reason for enhanced BUNV infection induced by high [K^+^]. We assessed the temporal expression of BUNV-N with viruses treated at pH 6.3 in high [K^+^] vs low [K^+^]. Fig 1F shows that the high [K^+^] virus expressed BUNV-N to detectable levels at 12 hours post infection (hpi), whilst the levels of the low [K^+^] virus were undetectable until 15 hpi, at which time BUNV-N expression remained reduced compared to high [K^+^] viruses. By 21 hpi both high and low [K^+^] viruses had reached comparable steady state levels of BUNV-N, suggesting that the high [K^+^]/pH 6.3 virus was able to establish an infection more quickly than the low [K^+^] virus, possibly due to its ability to more readily escape the endosomal system. The enhanced rates of infection displayed by high [K^+^]/pH 6.3 viruses were not due to plasma membrane mediated fusion since neither high nor low [K^+^]/pH 6.3 viruses displayed infectivity via traditional acid bypass assays [11] that involve pH induced fusion at the cell surface (S1 Fig). In addition, both high and low [K^+^]/pH 6.3 viruses could not establish an infection when added to cells in media supplemented with NH_4_Cl (to prevent infection via endosomes) further confirming endocytic entry for high [K^+^]/pH 6.3 viruses (S1 Fig).

Finally, we reasoned that if [K^+^] was related to the BUNV dependence on cellular K^+^ channel activity, that high [K^+^] treated viruses would be less sensitive to cellular K^+^ channel modulation. To investigate this, we assessed high vs low [K^+^] pH 6.3 treated viruses infected onto cells treated with the broad spectrum K^+^ channel inhibitor Tetraethylammonium (TEA), shown previously to inhibit BUNV infection [12]. High [K^+^] treated BUNV displayed reduced sensitivity to TEA treatment, which completely inhibited the infection of low [K^+^] BUNV (Fig 1G). We ruled out any role for K^+^ channel(s) in the virion, as when TEA was added *in vitro* during high [K^+^]/pH 6.3 treatment (and subsequently diluted in media prior to infection), it did not inhibit the rise in BUNV infection (Fig 1H). Taken together, these data strongly suggest that BUNV dependence on K^+^ channels is due to a requirement for high [K^+^] during virus entry.

### Production of infectious dual-labelled BUNV to track virus infection

**Fig 2:**
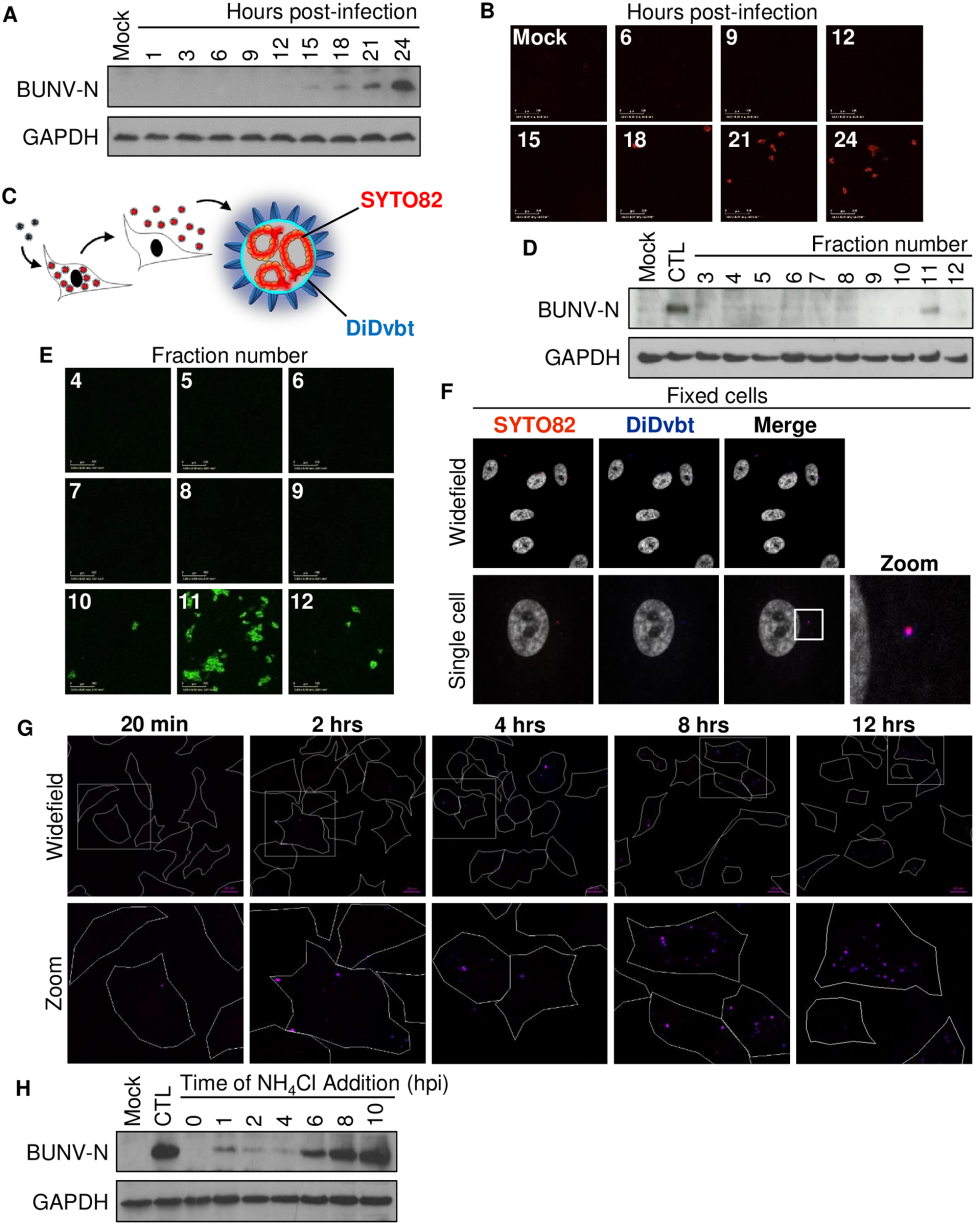
Production of SYTO82/DiD-BUNV to monitor virus trafficking. **(A)** Time course of A549 cells infected with BUNV. Cells were lysed at 3 hr intervals post-infection. Western blot analysis of BUNV-N protein and GAPDH (loading control) are shown (n = 3). **(B)** A549 cells were infected with BUNV supernatants collected from infected A549 cells at the indicated 3 hr intervals. Infected cells were fixed at 18 hpi and BUNV-N protein was labelled using anti-BUNV-N antibodies alongside Alexa-Fluor 594 nm secondary antibodies. Widefield images taken on the IncuCyte Zoom are shown (n = 3). Scale bar = 200μm **(C)** Schematic representation of BUNV labelling. A549 cells were infected with BUNV for 18 hrs, then SYTO82 dye was added to label the viral RNA segments until virus supernatants were collected at 24 hrs. Virus supernatants were concentrated, the BUNV envelope labelled with DiDvbt and SYTO82/DiD-BUNV was purified on a 10–30% iodixanol gradient. 1 ml fractions were collected (n = 3). **(D)** Fractions from SYTO82/DiD-BUNV purification were used to infect A549 cells for 18 hrs. Western blot analysis for BUNV-N was performed to confirm virus infectivity. **(E)** Cells were infected as in ***D***, fixed, and stained with anti-BUNV-N and Alexa Fluor-488 antibodies. Widefield images were taken on the IncuCyte Zoom (n = 3). Scale bar = 200 μM. **(F)** A549 cells were infected with SYTO82/DiD-BUNV for 2 hrs and fixed. SYTO82 (em._max_ 560 nm) and DiDvbt (em._max_ 665 nm) fluorescent signals were imaged alongside DAPI in fixed cells. **(G)** Cells were infected with SYTO82/DiD-BUNV for 1 hr at 4°C, then heated to 37°C and infection was allowed to proceed until fixing at 2 hrs, 4 hrs, 8 hrs or 12 hrs. Biotinylated EGF-488 (2 μg/ml) was added for 15 min at 37°C prior to fixing to act as a cell marker. Confocal images were taken for n>80 cells for each time point and the EGF-488 fluorescence channel was removed in the representative images (Scale bar = 10 μM). **(H)** NH_4_Cl (10 μM) was added at the indicated timepoints post-BUNV infection and BUNV-N expression assessed by western blot analysis 24 hours post-infection. CTL = no drug included during the timecourse (n = 3).

Given these data, we postulated that BUNV must encounter endosomes containing high [K^+^] during virus entry. To examine the entry of BUNV virions into cells, we applied dual labelling methodology previously described for dengue virus, chikungunya virus, and IAV [15–17]. The dual labelling strategy was employed as it allows more reliable discrimination between complete infectious particles and subviral complexes. This powerful strategy comprises a dual labelled BUNV in which the genomic RNA segments (vRNAs) are labelled with the cell permeant nucleic acid stain SYTO82 (excitation max = 540 nm, and emission max = 560 nm) by addition to cells 18–24 hpi, the time at which we observed the initiation of BUNV-N synthesis (and thus virus replication) and the release of infectious virus progeny (Fig 2A and 2B). During this timeframe we collected and purified released SYTO82-BUNV and then labelled the BUNV envelope with a spectrally distinct fluorophore DiDvybrant (excitation max = 648 nm, and emission max = 670nm) (Fig 2C). SYTO82/DiD-BUNV was then purified by fractionation and infectivity confirmed by western blot and immunofluorescent analysis in cells infected with specific SYTO82/DiD-BUNV fractions (Fig 2D and 2E). Importantly, SYTO82/DiD-BUNV was comparable to WT virus in terms of its infection kinetics or viral titres confirming the labelling process did not interfere with BUNV infectivity (S2 Fig). Confocal microscopy of cells infected with infectious SYTO82/DiD-BUNV allowed us to visualize internalised virions (Fig 2F). After 2 hrs, virus particles containing SYTO82 and DiDvbt signals were present, which showed complete colocalization. Using line scan analysis, the fluorescent signals were quantified by distance, which identified a peak of intense fluorescence that overlapped in the SYTO82 and DiDvbt channels (S2 Fig). This showed that the SYTO82/DiDvbt signals spatially colocalised, suggesting both fluorphores were incorporated into individual virus particles and that SYTO82/DiD-BUNV had endocytosed into cells in an intact form [3,18]. In addition to A549 cells, we were able to infect other BUNV susceptible cell lines with SYTO82/DiDvbt BUNV and show internalised colocalised fluorescent signals in the SYTO82 and DiDvbt channels (S2 Fig). Fig 2G shows that when added to cells and incubated at 37°C, a low number of labelled viruses were detectable entering cells at early timepoints (t = 20 mins). However, the number of internalised virions dramatically increased over time, most notably between 2–8 hrs. Our interpretation of these results is that the virus particles are endocytosed in a rapid and efficient manner, but the internalized viruses then accumulate in an endocytic compartment prior to endosomal escape. To confirm this observation, we performed experiments in which cells were infected in media supplemented with NH_4_Cl (to prevent infection via endosomes) added at defined timepoints post-infection (Fig 2H). When NH_4_Cl was added 1–4 h post infection (hpi), BUNV-N expression was drastically reduced (Fig 2H). BUNV-N expression began to recover when NH_4_Cl treatment was delayed to 6 hpi, and was unaffected when NH_4_Cl treatment was initiated at 8 hpi. This suggests that BUNV passage through endosomes takes up to 6–8 hrs in the A549 cells investigated in this study. Taken together, these data demonstrate the successful labelling of BUNV virion internal and external components to monitor the movement of intact virus particles during virus infection.

### BUNV traffics through endosomes containing high [K^+^]

**Fig 3:**
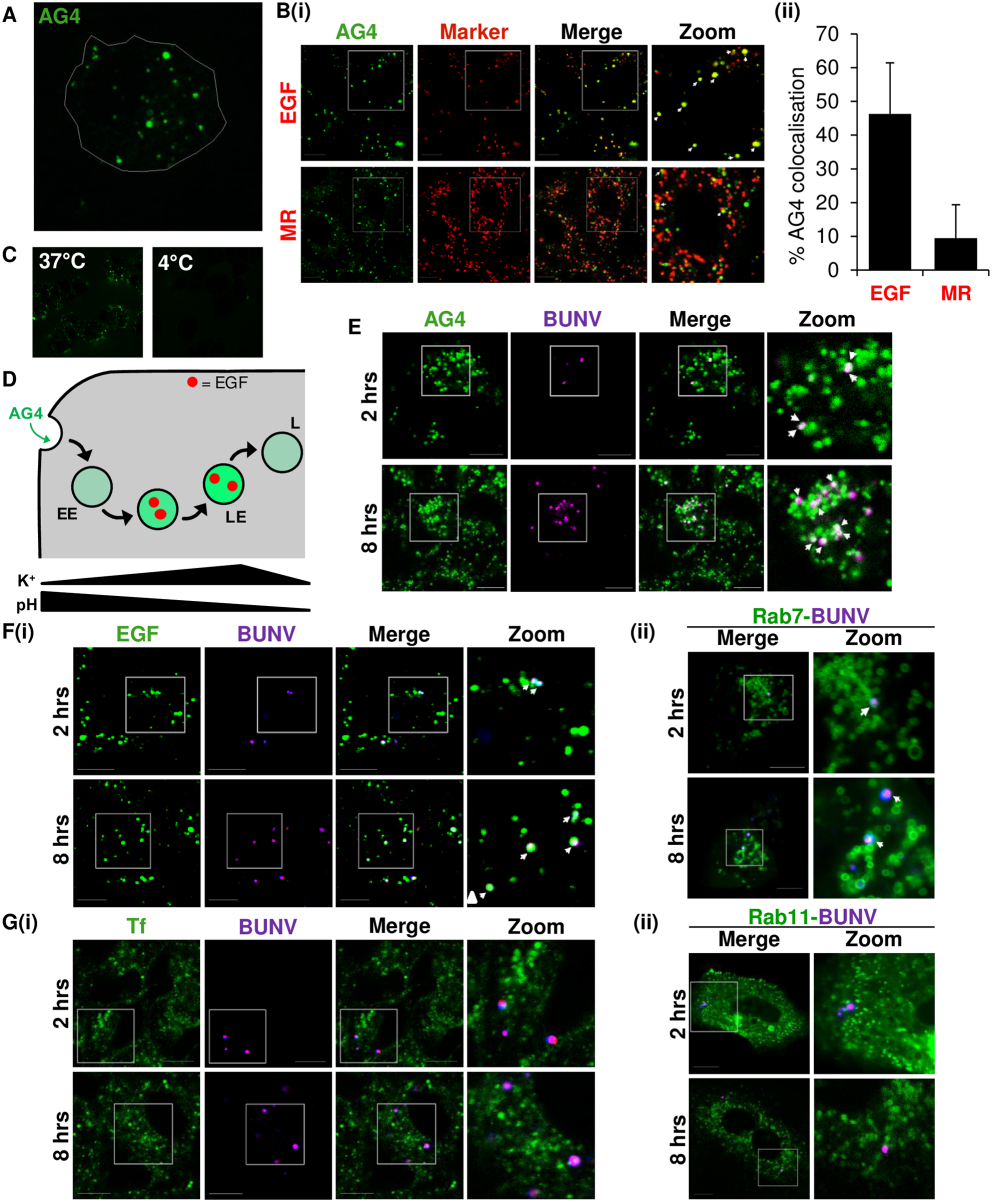
BUNV traffics through endosomes containing K^+^ ions. **(A)** AG4 (10 μM) was added to A549 cells for 40 min to allow endosomal uptake, alongside **(B)(i)** Texas Red labelled EGF (2 μg/ml) or Magic Red cathepsin B dye. Non-internalised dyes were subsequently removed and live cells were imaged. Representative images are shown (n≥100 cells). Scale bar = 10 μM. **(ii)** Total numbers of AG4 positive puncta were counted per cell and % of colocalised AG4 puncta with each marker calculated in ≥100 cells. **(C)** AG4 (10 μM) was added to A549 cells for 40 min at either 37°C or 4°C and live cells were imaged as in A. Scale bar = 10 μM. **(D)** Schematic representation of AG4 uptake into endocytic vesicles and increased fluorescence with passage through early endosomes (EE) into late endosomes (LE) identifying K^+^-rich regions, identifiable using Texas Red labelled EGF. AG4 fluorescence decreases with passage into lysosomes (L). **(E)** A549 cells were infected with labelled-BUNV in the presence of AG4 (10μM) to allow virus penetration into cells and live cells were imaged 2 hrs or 8 hrs post-infection. Images are representative of ≥ 50 cells. **(F)(i)** A549 cells were infected with SYTO82/DiD-BUNV and EGF-488 (2 μg/ml) for 1 hour at 4°C and cells warmed to 37°C for the indicated timepoints. Images were taken of live cells at the indicated time points post-warming and are representative of ≥60 cells. Scale bar = 10 μM. **(ii)** Cells were transfected with Rab7-GFP and infected as in **F(i)** 24 hours post transfection. Images are representative of ≥ 40 cells. Scale bar = 10 μM. **G(i)** as in **F(i)** but cells were infected in the presence of 488-labelled Tf (25 μg/mL) or **(ii)** cells transfected with Rab11-GFP.

We next sought to investigate if BUNV traffics through K^+^ containing endosomes. To label K^+^ containing vesicles, we used the membrane-impermeable K^+^-specific fluorescent probe Asante Potassium Green-4 (AG4), the membrane permeable form of which has previously been used to monitor cytoplasmic [K^+^] [19]. When cells were imaged following AG4 addition, bright fluorescent puncta were observed confirming the presence of K^+^ ions in endosomal compartments (Fig 3A). Vesicles displaying the most intense AG4 fluorescence were predominantly found to colocalize with internalised Epidermal Growth Factor (EGF) (45 mins EGF treatment, Fig 3B(i) and 3B(ii)) which would be predicted to have traversed EEs to LEs at this time point [20], confirming that LEs are the predominant sites of AG4 fluorescence [11] and herein referred to as high K^+^ containing endosomes. In our experiments, AG4 fluorescence and thus areas of high endosomal K^+^ rarely extended to lysosomal compartments, and as in live cells stained with magic red [21], minimal AG4 colocalisation was evident at all timepoints of AG4 labelling investigated (Fig 3B(i) and 3B(ii), S3 Fig). We further confirmed that AG4 was specifically labelling endocytic vesicles as at low temperatures non-permissive to endocytosis, no AG4 uptake was observed (Fig 3C). Taken together, these data suggested that [K^+^] increases with passage along the endocytic pathway, peaking in LEs, and then decreasing upon endosome maturation into lysosomes (depicted in Fig 3D).

As [K^+^] speeds up BUNV infection kinetics, we assessed whether BUNV traffics through high [K^+^] containing endosomes. Cells were infected with labelled-BUNV at 37°C in the presence of AG4 for 2 or 8 hrs and the trafficking of BUNV in live cells assessed. Fig 3E shows representative images of ≥50 cells that demonstrate the consistent detection of SYTO82/DiDvbt BUNV in distinct puncta of high AG4 fluorescence. These vesicles represent the late endosomal compartments of high [K^+^] identified in Fig 3B, as SYTO82/DiDvbt BUNV was also found to accumulate in EGF/ Rab7 positive endosomes at identical timepoints (Fig 3F(i) and 3F(ii), S4 Fig). Co-localisation with internalised Transferrin (Tf) or Rab11 (markers of early/recycling endosomes) at 2 hpi was however, minimal (Fig 3G(i) and 3G(ii)). BUNV virions did enter EGF/Tf positive vesicles at earlier timepoints post-penetration (t = 20) (S5 Fig), but by 1 hpi a loss of BUNV-Tf co-trafficking was observed. These data suggested that BUNV is trafficked with EGF from EE-LEs (≥ 1 hpi), since EEs are the only compartment at which internalised EGF and Tf co-localise. Taken together, these data suggest that invading BUNV accumulates in LEs where it encounters the high [K^+^] required for the initiation of virus infection.

### Modulation of K^+^ channels influences K^+^ distribution across the endosomal network

**Fig 4:**
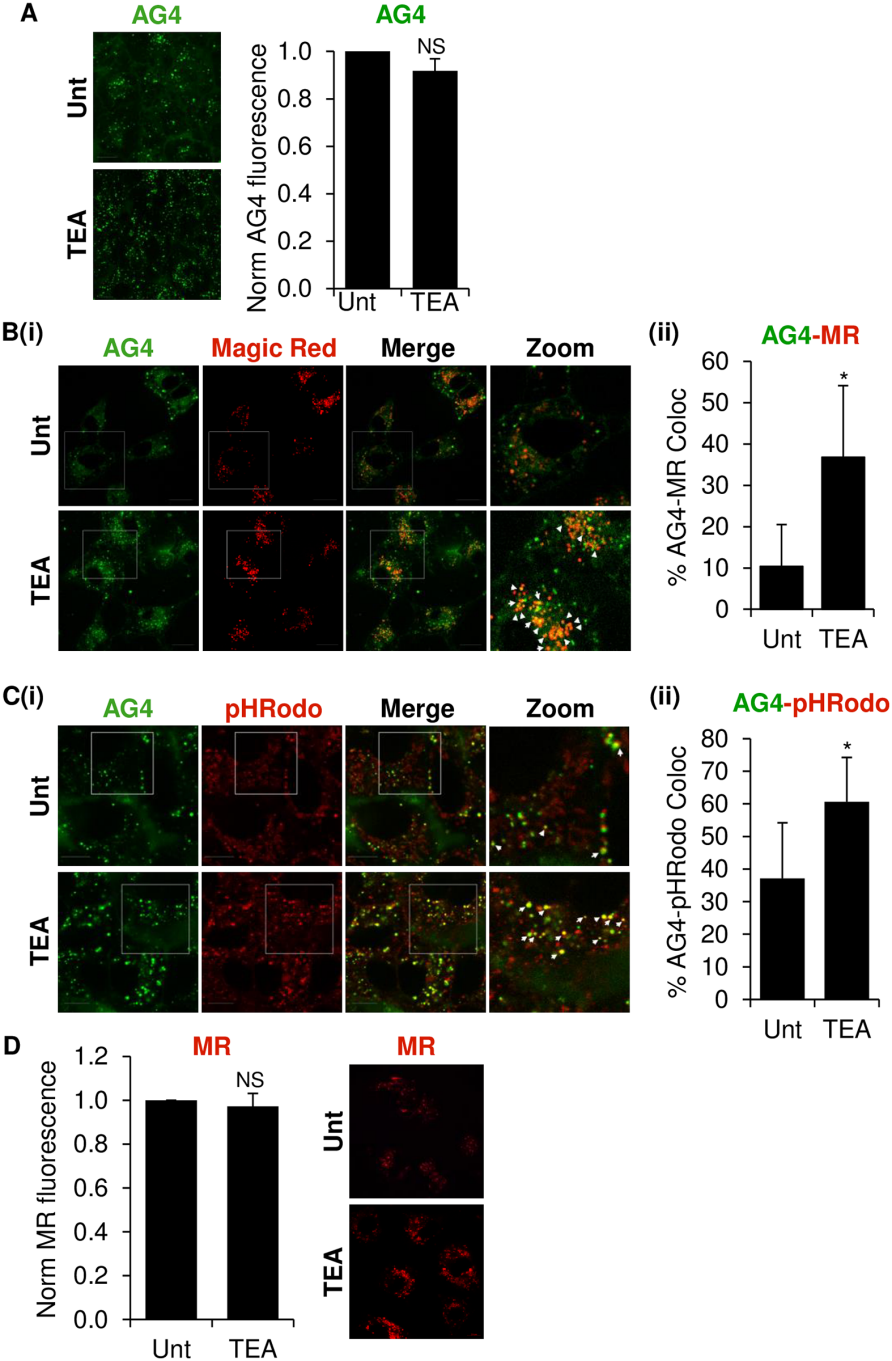
K^+^ channel modulation can impede normal K^+^ accumulation across the endocytic network. **(A)** A549 cells were treated for 30 min with 10 mM TEA or left untreated. AG4 (10 μM) was then added in the presence or absence of drug for 40 min. Dye was removed and TEA was re-added onto cells. Fluorescence intensities were quantified using IncuCyte ZOOM imaging and analysis software, and data normalised to untreated (unt) controls over three independent cell populations. NS–no significant difference between no-drug and TEA treated controls (p≥0.05). Scale bar = 10 μM. **(B) (i)** Cells were treated with 10 mM TEA (or left untreated) and AG4 (10 μm) added as in ***A***, with the addition of Magic Red during the 40 min incubation with AG4. Representative images are shown (n≥60 cells). Scale bar = 10 μM. **(ii)** Total number of AG4 positive puncta were counted per cell ± TEA and the % of colocalised puncta presented. n≥60 cells, (* = p≤0.05). Scale bar = 10 μM. **(C) (i)** Cells were treated with 10 mM TEA or left untreated, and AG4 (10 μM) added as in ***A***, with the addition of the pH indicator pHrodo red dextran (10 μg/ml) during the 40 min incubation with AG4. Representative images are shown (Scale bar = 10 μM) and the % of co-localised puncta presented in **(ii)** n≥60 cells (* = p≤0.05). **D** Fluorescence intensity of Magic Red was quantified using IncuCyte ZOOM imaging and analysis software and data normalised to untreated controls over three independent cell populations. NS–no significant difference between untreated and TEA treated cells (p≥0.05). Representative images are also shown.

Whilst K^+^ channels control the selective passage of K^+^ ions across the plasma membrane, their role in endosomal K^+^ accumulation has not been fully defined. Owing to the effects of high [K^+^] on BUNV infectivity demonstrated in Fig 1, and the effects of K^+^ channel modulators on BUNV endosomal processes, we sought to assess if the K^+^ concentration within compartments of the endocytic pathway are influenced by K^+^ channel blockade. To investigate this, AG4 fluorescence was assessed in cells treated with the K^+^ channel inhibitor TEA. Upon TEA addition, the overall intensity of AG4 staining was unaffected when quantified per cell (Fig 4A). AG4 was also still evident in vesicular structures throughout the cytoplasm in the face of K^+^ channel modulation (Fig 4A). Interestingly however, when the localisation of AG4 fluorescence was investigated ± TEA, AG4 was found to redistribute and significantly colocalise with magic red positive lysosomes in TEA treated cells (~9.41% colocalisation in no-drug cells vs ~36.78% colocalisation plus TEA, Fig 4B). Our interpretation of these data is that areas of high endosomal [K^+^] had shifted from late endosomes into lysosomes in the face of K^+^ channel inhibition, suggesting that K^+^ channel modulation disrupted the normal distribution of K^+^ across the endosomal system. To confirm this finding, we assessed the localisation of AG4 fluorescence ± TEA with respect to pHrodo dextran, a pH-sensitive fluorophore that increases in intensity with increasing endosome acidity encountered through the endocytic pathway. Fig 4C shows that in no-drug cells, AG4 showed ~37.09% overlap with acidic endosomes, whilst in the presence of TEA, AG4 was found to re-localise to areas of intense pHrodo fluorescence (~60.60% co-localisation with pHrodo), confirming that AG4 fluorescence had shifted to more acidic lysosomal compartments (Fig 4C). Importantly, in the presence of TEA no effects on the distribution or intensity of magic red staining were observed (Fig 4D) confirming that TEA does not interfere with endosomal pH or normal lysosomal function. Taken together, these data demonstrate that K^+^ channel modulation can influence the distribution of K^+^, across the endosomal network.

### K^+^ channel modulation interferes with BUNV endocytic processes

Given these data, we reasoned that altering endosomal [K^+^] balance with K^+^ channel blockade would influence BUNV movement through the endosomal system.

**Fig 5:**
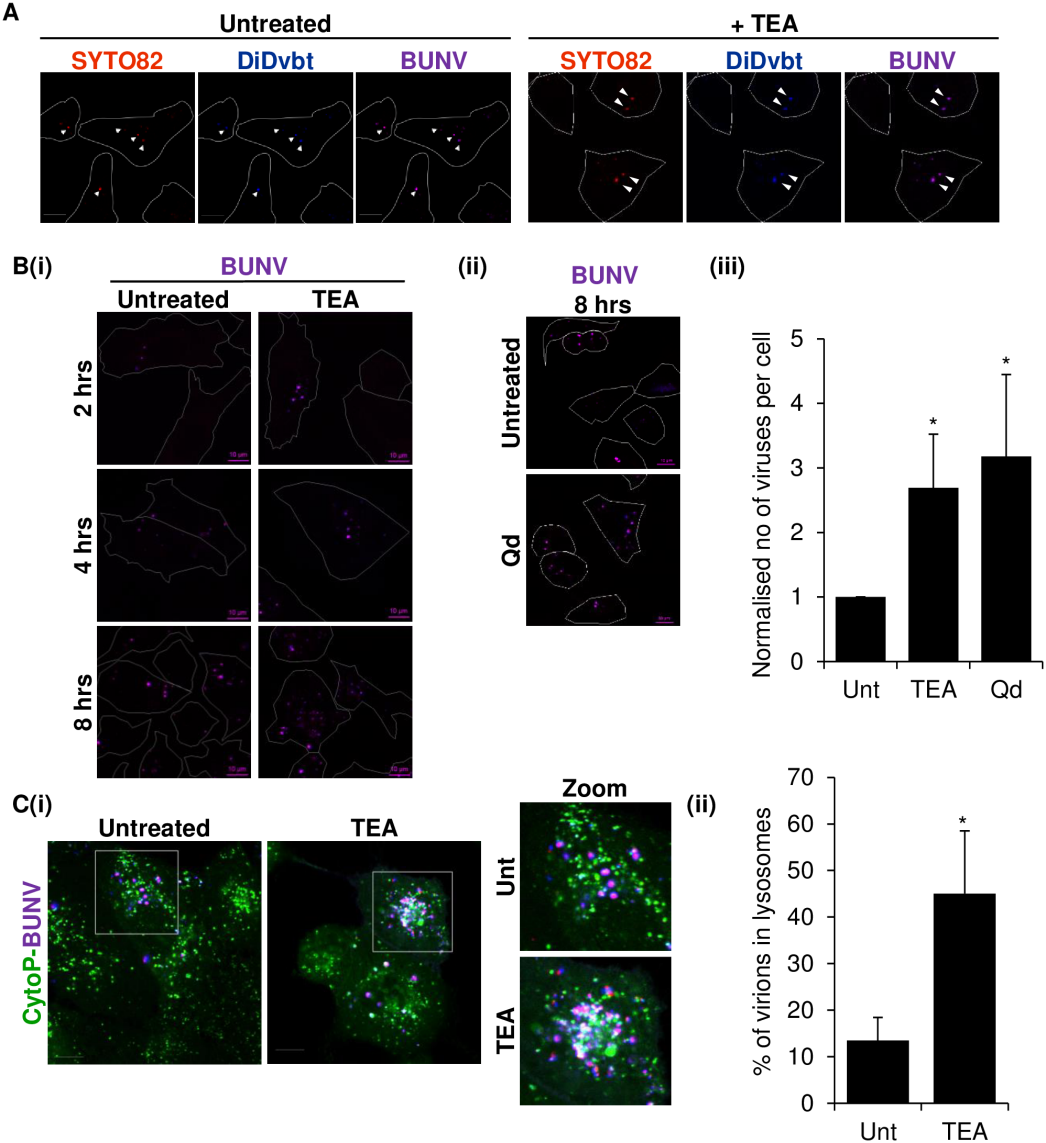
K^+^ channel modulation arrests BUNV trafficking in endosomes. **(A)** Cells were treated with TEA (10 mM) for 30 min (or left untreated) and infected with SYTO82/DiD-BUNV for a further 4 hrs in the presence/absence of TEA. EGF-488 was added for the final 15 min of infection and cells were fixed 4 hpi. Confocal images were taken and the EGF-488 fluorescence channel removed in the representative images showing only SYTO82 and DiDvbt (n≥40). Scale bar = 10 μM. **(B)(i)** Cells treated with TEA (10 mM) or left untreated as in ***A***, were infected with SYTO82/DiD-BUNV and fixed at 2, 4 or 8 hpi. EGF-488 (2 μg/ml) was added for 15 min prior to fixation as in **A**, with the representative images showing only SYTO82 and DiDvbt channels (n>65 cells). Scale bar = 10 μM. **(ii)** As in ***(i)*** but cells were treated with Qd (200 μM) and fixed 8 hpi (n>65 cells). **(iii)** The number of SYTO82/DiD-BUNV virions per cell were quantified using images from **(i)** and **(ii)** for n>65 cells and normalised to the untreated (no-drug) control. **(C)** A549 cells were infected with SYTO82/DiD-BUNV for 1 hour at 4°C and treated with cytopainter to label lysosomes. Cells were warmed to 37°C for 1 hr, virus/dye removed by washing and cells incubated for up to 8 hpi. Representative live cell images are shown (≥80 cells). Scale bar = 10 μM. **(ii)** The number of SYTO82/DiD-BUNV virions co-localising with cytopainter positive puncta were calculated and the % of co-localised puncta presented in **(ii)** (* = p≤0.05).

To examine this, cells were infected with SYTO82/DiD-BUNV in the presence of TEA and the penetration of SYTO82/DiD-BUNV into cells assessed. Fig 5A clearly identified the presence of intact internalised SYTO82/DiD-BUNV within the cell cytoplasm suggesting that BUNV penetration into cells does not depend on K^+^ channel activity, consistent with our previous findings [12]. However, when SYTO82/DiD-BUNV was added to cells for various times up to 8 hpi in the presence of TEA, we observed more SYTO82/DiD-BUNV particles visible inside cells compared to no-drug cells (Fig 5B(i) and 5B(iii)). When the number of resident SYTO82/DiD-BUNV virions were quantified in no-drug vs TEA treated cells, this observation was confirmed as TEA consistently enhanced the number of cell resident virions (2.7 fold increase in number of virions per cell) suggesting that altering [K^+^] levels in the endo-lysosomal system, acted to arrest BUNV trafficking. These data were confirmed with an independent broad spectrum K^+^ channel modulator quinidine (shown previously to inhibit BUNV [12]) that led to a ~3.1 fold increase in the number of BUNV virions per cell 8 hpi (Fig 5B(ii) and 5B(iii)). This suggested that K^+^ channel inhibitors interfere with BUNV endosomal trafficking and impede endosomal release, most likely through their influence on endosomal [K^+^] distribution.

We reasoned that in the presence of K^+^ channel blockade, BUNV would encounter the high [K^+^] required for the establishment of BUNV infection only in the acidic environment of lysosomes (pH ≤5.3). We confirmed this as BUNV was found to accumulate in cytopainter (a lysotropic dye) positive lysosomes in the presence of TEA, whilst untreated cells rarely demonstrated BUNV trafficking into lysosomal compartments (Fig 5C(i) and 5C(ii)), ~13.48% co-localisation in no-drug cells vs ~45.06% co-localisation in TEA treated cells n≥50 cells). Since we previously demonstrated BUNV virions are non-infectious at pH ≤5.3 (Fig 1), lysosome resident virions in TEA treated cells would be expected to be non-infectious. This provides the first mechanistic insight into why K^+^ channel inhibition prevents a viral infection [12].

## Discussion

We previously identified a requirement for host cell K^+^ channels, which are found in all cell types, early during BUNV infection and that this dependence is shared across other bunyaviruses [12]. The stage of infection affected was predicted to include virion trafficking through the endocytic network, fusion of the viral and host membranes releasing RNPs into the cytoplasm, and RNP trafficking to viral factories within the Golgi [4,5,7,8]. Our knowledge of these processes and the host factors involved were lacking and why a K^+^ channel would contribute to such processes was unclear. Here, through the production of a novel dual labelled BUNV, we now demonstrate how K^+^ channels can directly influence virus infectivity through the control of endosomal K^+^ balance, as the K^+^ ion has direct effects on the BUNV virion.

### Ionic balance and pH changes can enhance BUNV infectivity

Viruses require different triggers to aid endosomal escape [22]. For many enveloped viruses, including bunyaviruses, low pH elicits structural changes required for virion fusion, initiated by the interaction of structural proteins with host membranes [18,23]. In this study, treatment of BUNV virions at pH ~6.3 coupled to high [K^+^] upregulated the speed of virus infection (BUNV-N was detectable at 12 hpi as opposed to 15 hpi for low [K^+^] viruses) (Fig 1). It is unclear how K^+^ improves the speed of virus infection, but these data reveal that acidification is not the sole trigger required for the early stages of virus entry into the cytosol. In agreement with these findings, priming of IAV by *Stauffer et al* (2014) identified that pH <6.0 and high [K^+^], altered the interactions between IAV RNPs and Matrix protein (M1), required for efficient core destabilisation and virion uncoating. Whilst bunyaviruses lack a viral matrix protein, their Gn cytoplasmic tails interact directly with RNPs [23]. We therefore speculate that K^+^ may facilitate the uncoating of BUNV and release the RNPs from Gn binding, reminiscent of core destabilisation in IAV infection. This implies a necessity for BUNV virions to traffic through cellular vesicles rich in K^+^, which we demonstrated using our dual labelled BUNV (Fig 3).

An increase in BUNV infectivity was also generated by pre-treatment with high [Na^+^] (Fig 1). Na^+^ and K^+^ possess similar atomic radii and BUNV may not distinguish easily between these ions. This is contrast to IAV, where high [Na^+^] had no effect [11], suggesting that the ionic requirements of BUNV differ. In this regard, K^+^ influx into IAV virions is mediated by the M2 viroporin, which like cellular ion channels can select for K^+^ over Na^+^ owing to the selectivity of the channel pore [24]. BUNV has no known viroporin and we demonstrate the absence of a K^+^ channel in the virion (Fig 1H). However, ion binding to proteins can dictate numerous properties, such as surface tension, protein stability, protein–protein interactions and protein-lipid interactions [25]. Thus it can be speculated that a pH-mediated alteration of the BUNV virion surface, such as those previously shown to occur for the Gn and Gc glycoproteins, may facilitate K^+^ or Na^+^ entry into BUNV virions or expose domains in Gn and Gc that interact with these ions to promote virion destabilisation, enhancing BUNV infection. Importantly in our experiments, infection by pH 6.3/high [K^+^] treated-BUNV was less sensitive to inhibition by K^+^ channel blockers (Fig 1). This implicates the specific effects of [K^+^] for the dependence of BUNV on cellular K^+^ channels.

### Does K^+^ channel modulation influence endosomal K^+^ accumulation?

Recently, it has been identified that luminal ion concentrations are regulated across the endocytic network [10]. Whilst this ion movement is difficult to assess due to the internal localization and small size of endocytic compartments by classical techniques such as patch clamp analysis, it is known that multiple ion transporters, channels and exchangers function within endosomes to control the luminal ionic milieu; the dysfunction of which can lead to defects in pH control and lysosomal storage-disorders [10]. Multiple Cl^-^ channels have been identified within endosomes that increase luminal Cl^-^ concentrations through Cl^-^/H^+^ exchange. Other ion transporters and channels abundant in endosomes include the transient receptor potential Ca^2+^ channels, Na^+^/H^+^-exchangers and the two-pore Ca^2+^ channels, shown previously to be required for Ebola virus endosome escape [10,26]. Interestingly pioneering work by Cang et al 2015 identified TMEM175 as a new K^+^ channel that is responsible for K^+^ conductance in endosomes and lysosomes [27]. However since the pharmacological properties of TMEM175 do not align with the K^+^ modulating compounds that inhibit BUNV (not inhibited by TEA or quinine) we speculate the involvement of other TEA K^+^ sensitive channels. We speculate that TEA molecules may reside near the membrane during virus entry and are captured into BUNV containing vesicles that internalise into endosomal compartments. TEA uptake and endosomal accumulation has been previously reported [28]. The presence of TEA in these vesicles inhibits endosomal K^+^ channels, slowing K^+^ flux from the cytoplasm into endosomes down its electrochemical gradient. Invading BUNV might then encounter endosomes of high [K^+^] at more acidic pH (lysosomal pH can range from 6.0–4.9), conditions under which BUNV virions were demonstrated to be non-infectious in biochemical assays (Fig 1). Accordingly we observed the accumulation of BUNV in lysosomes in TEA treated cells (Fig 5C). This provides mechanistic insight into the inhibition of virus infectivity through K^+^ channel modulation and identifies a previously undescribed role of a TEA sensitive K^+^ channel in the regulation of endosomal K^+^ concentrations, and thus endosome function (S6 Fig). Interestingly, our previous studies suggest that the K^+^ channel family required by BUNV are K_2P_ channels [12], known to localise to endocytic compartments [29]. K_2P_ channels are regulated by a multitude of signals important for endosome integrity, including pH and membrane stretch [30]. Our current work seeks to identify an endosomal role for K_2P_ channels by identifying the specific channel family member(s) required by BUNV, implicating their functionality in endosomal K^+^ balance. In this regard it is notable that some K_2P_ channels can pass both K^+^ and Na^+^ in response to changes in pH, low [K^+^] and sequence variation [31] and we show that both these ions can enhance BUNV infectivity (Fig 1). These experiments are ongoing and as the 15 members of this channel family can heterodimerize to form new channels with distinct electrophysiological and pharmacological properties, they may prove complex [32]. Our proposed endosomal role of K_2P_ channels aligns well with recent K_2P_ silencing studies that suggest a loss of K_2P_ function does not impair plasma membrane polarity [33], implying an intracellular role for K_2P_ channel functionality.

## Materials and methods

### Cells and virus

A549 (human alveolar carcinoma epithelial) and SW13 (human adrenal carcinoma) cells were obtained from the European Collection of Cell Cultures (ECACC) and maintained in a humidified incubator at 37°C with 5% CO_2_. Cells were cultured in Dulbecco’s modified eagle’s medium (DMEM, Sigma) supplemented with 10% fetal bovine serum (FBS), 100 U/ml penicillin and 100 μg/ml streptomycin (1% pen/strep). Wild type BUNV stocks were made from clarified infected cell supernatants and titre estimated by plaque assay yielding ~2.9x10^7^ PFU/ml.

### Infection assays

A549 cells were seeded into 6 well plates (~3x10^5^ cells per well) and allowed 24 hrs to adhere. Cells were infected with BUNV (MOI = 0.1) in complete media and infection was allowed to proceed for 18 hrs (unless otherwise stated) before cell lysis or fixation.

### Timecourse of BUNV-N expression

Cells were infected with BUNV (MOI = 0.1) for 1 hr to allow virus entry. Media and non-internalised virions were removed (1 hpi) and replaced with fresh media. At 3 hr intervals from 1 to 24 hpi, cells were lysed and the presence of BUNV-N protein determined by western blot analysis.

### Timecourse of NH_4_Cl addition

Cells were infected with BUNV (MOI = 0.1) for 1 hr to allow virus entry. NH_4_Cl (10 mM) was included in the media during the 24 h infection period (T = 0), or added at the indicated timepoints from 0–10 hpi, then incubated for 24 hrs. The presence of BUNV-N protein was determined by western blot analysis.

### SYTO82/DiD-BUNV production and purification protocol

A549 cells were seeded into 2 x T175 cm^2^ flasks and incubated at 37°C for 24 hrs to adhere. Cells were infected with BUNV (MOI = 0.5) in optimem for 3 hrs at 37°C, with gentle rocking. Optimem was then removed and replaced with low serum DMEM media containing 2% FBS. At 18 hpi, the nucleic acid dye SYTO82 (Thermo Fisher Molecular Probes) was added directly into the media at a final concentration of 2.5 μM. Infected cells were incubated for a further 6 hrs (24 hpi) and the supernatant containing released SYTO82-BUNV virions collected. Supernatants were spun at 375 x *g* for 10 min to pellet the cellular debris and the virus-containing media collected. Clarified supernatants from both flasks were combined in a 250 ml sterile glass bottle with a sterile stirrer bar. 50% (w/v) poly (ethylene glycol) (PEG) 6,000 (in TNE buffer (0.01 M tris HCl, 0.1 M NaCl, 1 mM EDTA, pH 7.4); Fluka Analytical) was added to the supernatant at a final concentration of 10% (w/v) PEG. The virus-PEG solution was placed on a low stir at 4°C overnight and spun at 3,000 x *g* for 40 min at 4°C. Media supernatant was removed and virus-PEG precipitate was re-suspended in TNE. The prep was concentrated on a double iodixanol (optiprep density gradient medium; Sigma) cushion column, containing a 10% iodixanol fraction over a 60% iodixanol fraction. The cushion was loaded into SW40 Ti rotor buckets (Beckman Coulter) and spun in an ultra-centrifuge at 160,000 x g for 90 min at 4°C (maximum acceleration and slow deceleration). Virus was collected from the 10%-60% iodixanol interface. The collected virus was labelled using the fluorescent DiD Vybrant cell-labelling solution (DiDvbt; Thermo Fisher Molecular Probes), which labels the viral lipid membranes, by incubation in 5 μM DiDvbt for 2 hrs at 4°C with gentle rocking. SYTO82/DiD-BUNV was loaded onto an iodixanol gradient column, formed with fractions of 30%, 25%, 20%, 15% and 10% iodixanol. Virus was purified by ultra-centrifugation at 250,000 x *g* for 90 min at 4°C (maximum acceleration and no brake deceleration). 1 ml fractions were collected (stored at -80°C) and A549 cells were infected with each fraction for 18 hrs to confirm the presence of infectious virus by western blot and immunofluorescent staining.

### Plaque assays

SW13 cells were seeded into 12-well plates and BUNV infections were performed once cells reached 90–95% confluency. Fractions collected during SYTO82/DiD-BUNV purification were used to generate a 10^−2^ dilution of each fraction in serum free media. Dilutions were infected alongside a WT BUNV control (MOI = 0.1) onto SW13 cells for 1 hr at 37°C, after which media was removed and replaced with a carboxy-methyl cellulose (CMC, Sigma) overlay—1.6% (w/v) high viscosity CMC diluted in an equal volume of complete media. Cells were incubated for 6 days and fixed with 20% formaldehyde by addition directly into the CMC/media overlay, for 1 hr at 4°C. Cells were then washed with dH_2_O and stained using 0.1% crystal violet solution (Sigma) for 15 min. Cells were finally washed with dH_2_O and the number of plaques counted to estimate viral titre.

### Assessment of virus release

A549 cells were infected with BUNV (MOI = 0.1) and media replaced 1 hr post-virus entry (1 hpi). Supernatants were collected at 3 hr intervals from 3 hpi to 24 hpi and stored at ^-^80°C. Supernatants were used to directly infect new A549 cells and infection allowed to proceed for 18 hrs prior to fixing cells in 4% paraformaldehyde. Immunofluorescent staining was performed to label the BUNV-N protein and IncuCyte ZOOM live cell imaging used to determine the number of infected cells.

### Western blot analysis

At 18 hpi (unless otherwise stated) cells were lysed using a Leeds lysis buffer (LLB; 25 mM glycerol phosphate, 20 mM tris, 150 mM NaCl, 1 mM EDTA, 1% triton x 100, 10% glycerol, 50 mM NaF, 5 mM Na_4_O_7_P_2_, pH 7.4) supplemented with protease inhibitor cocktail (Thermo Scientific), for 15 min at 4°C. Lysates were collected and run on 15% sodium-dodecyl sulphate (SDS) gels using SDS-PAGE. Samples were transferred onto polyvinylidene difluoride (PVDF; Millipore) membranes using a Bio-Rad Trans-blot Turbo. Membranes were blocked in 10% milk in PBS-tween for 1 hr. Proteins were labelled with primary antibodies overnight at 4°C, then with corresponding secondary antibodies for 1 hr. Labelling was detected using the chemiluminescence (ECL) system and film exposed using a Xograph processor. Results were scanned and cropped to antibody bandwidths.

### BUNV pH/ion assays

BUNV (2 μl; MOI = 0.1) was incubated at 37°C for 2 hrs in the presence of a high-salt buffer (20 μl; 1:11 dilution of BUNV) at pH 7.3, 6.3 or 5.3. Control experiments were carried out by incubation in phosphate buffered saline (PBS) or buffers at pH 7.3, 6.3 or 5.3, without high-salt addition. The high-salt and low pH were subsequently diluted by addition of 1 ml DMEM and immediately added to A549 cells. Infection was allowed to proceed for 18 hrs before lysis. In experiments where the K^+^ channel inhibitor Tetraethylammonium (TEA, Sigma) was applied to cells, cells were pre-treated with drug for 30 min prior to addition of the pH/ion treated BUNV. The drug remained present throughout infection. To test for the presence of K^+^ channels in the BUNV virion, BUNV was pre-treated with buffer at pH 6.3 containing low/high potassium chloride (KCl) and TEA (10 mM) for 2 hours which was diluted in media prior to infection.

### pH/ion buffers

Buffers were prepared containing 20 mM tris for pH 7.3, 30 mM bis-tris for pH 6.3 and 50 mM sodium-citrate for pH 5.3. The desired high-salt concentrations were achieved by addition of 5–140 mM KCl or 140mM sodium chloride (NaCl) (mimicking intracellular concentrations) or 140mM K_2_SO_4_. Buffers were made and adjusted to the desired pH (on the day of the experiment) using hydrochloric acid for tris and bis-tris buffers, and citric acid for sodium-citrate buffers.

### Acid-bypass assay

Pre-infection priming of the BUNV virions was carried out using the pH 6.3 (-K+) CTL buffer and the pH 6.3, high K+ buffer. Primed virions were added to A549 cells for 1.5 hrs on ice, followed by a wash step with cold DMEM to remove unbound virions. Acid-bypass to induce virion fusion at the plasma membrane was then carried out following the procedure outlined by *Stauffer et al 2014*. Warm pH 5.0 fusion buffer (DMEM containing 50 mM sodium citrate) was added to cells for a 2 min pulse at 37°C, alongside control samples incubated with either an acid-bypass control buffer (DMEM, 50 mM HEPES, 20 mM NH4Cl, at pH 7.4) or DMEM alone. Cells were subsequently washed twice with cold DMEM, then warm acid-bypass control buffer was added to all except the DMEM alone control wells, where DMEM was re-added (allows endocytic entry of virus, to confirm successful virus priming). Infected cells were incubated for 17 hrs and lysed.

### Antibodies

As a marker for infection, sheep anti-BUNV-N primary antibodies generated in the JN Barr lab were used and subsequently labelled with horseradish-peroxidase (HRP) conjugated anti-sheep (Sigma) secondary antibodies. GAPDH was labelled with rabbit anti-GAPDH primary antibodies (Santa Cruz) and anti-rabbit (Sigma) secondary antibodies as a loading control, identifying equal protein concentrations loaded onto SDS gels.

### Immunofluorescent staining and IncuCyte zoom analysis

A549 cells infected with BUNV were fixed at 18 hpi with 4% paraformaldehyde (PFA) for 10 min at 4°C. Cells were then permeabilised with 0.1% triton-X100 in PBS for 10 min and blocked in 1% bovine serum albumin (BSA; Sigma) in PBS for 30 min. BUNV infection was detected with primary anti-BUNV-N antibodies, added to cells in 1% BSA for 2 hrs and washed in PBS (x4). The corresponding fluorescent Alexa-Fluor 488 nm or 594 nm conjugated anti-sheep (Invitrogen-Molecular Probes) secondary antibodies were added in 1% BSA for a further 2 hrs and cells were washed 4 times in PBS. Widefield images of 2.15 mm^2^ were taken using the IncuCyte Zoom imaging system to identify fluorescently-labelled infected cells. The number of fluorescent cells were quantified and normalised to controls (untreated WT BUNV infection).

### Confocal imaging

#### Fixed cell imaging of fluorescently labelled SYTO82/DiD-BUNV

24 hrs prior to treatment, A549 cells were seeded onto polylysine-coated glass coverslips and incubated at 37°C to adhere. The gradient fraction containing purified SYTO82/DiD-BUNV was added to cells (MOI = ~4) for 2 hrs at 37°C. 2 μg/ml conjugated epidermal growth factor (EGF)-488 (Biotinylated EGF, complexed to Alexa-Fluor 488; ex._max_ 490 nm, em._max_ 525 nm; Thermo Fisher Molecular Probes) was added 15 min prior to fixing, to be used as a cell marker. Cells were fixed in 4% PFA, washed with PBS and mounted onto slides using ProLong Gold reagent containing DAPI (Invitrogen). An inverted 510-META laser-scanning confocal microscope (Zeiss) was used with an oil-immersion 40x or 63x objective lens to image fluorescent labelling. Widefield images were taken and represent single optical sections of 50 μm thickness. The fluorophores used possessed differing excitation_max_ (ex._max_) and emission_max_ (em._max_) spectra, SYTO82 ex._max_ 540 nm and em._max_ 560 nm and DiDvbt ex._max_ 645 nm and em._max_ 665 nm, which do not significantly overlap and bleed-through was not observed. Colocalisation of fluorophores was analysed by line scan and fluorescence quantified at intervals across the cell (Zeiss Zen 2011 Software).

#### Live cell imaging—SYTO82/DiD-BUNV

A549 cells were seeded onto 9 cm^2^ glass-bottom dishes or 1 cm^2^ glass-bottom 8-well μ-slides (Ibidi) 24 hrs prior to infection. Cells were infected with SYTO82/DiD-BUNV (MOI = ~8) in optimem at 4°C for 1 hr, to allow virus binding to the cell surface. Conjugated EGF-488 (2 μg/ml) or TF-488 were added simultaneously during infection, in the presence or absence of 10 mM TEA or 200 μM quinidine as specified at 37°C for the indicated timepoints. For the assessment of endosomal K^+^, 10 μM Asante Green-4 dye (AG4; ex._max_ 490 nm, em._max_ 540 nm; TEF Labs) was added to cells alongside SYTO82/DiD-BUNV at 37°C for 1hr, and cells subsequently washed and incubated at 37°C for the indicated timepoints. To assess Rab7 and Rab11 co-localisation, cells were transfected 24 hours prior to SYTO82/DiD-BUNV infection. To determine BUNV movement into lysosomes, cytopainter (Life Technologies) was added to cells alongside SYTO82/DiD-BUNV at 4°C for 1 hr, cells were washed, and warmed to 37°C and imaging at the indicated timepoints. For all experiments, cells were imaged on an inverted 510-META laser-scanning confocal microscope for up to 8 hpi. Time series images were acquired at 20 second intervals to visualise virion entry and post-entry trafficking.

#### Live cell imaging—AG4

A549 cells were seeded onto 1 cm^2^ glass-bottom 8-well μ-slides and incubated for 24 hrs. Cells were treated with 10 μM AG4 and 2 μg/ml Texas Red labelled EGF, Magic Red cathepsin B dye (ImmunoChemistry Technologies; ex._max_ 590 nm, em._max_ 620 nm) or 10 μg/ml pHRodo Red Dextran (Life Technologies; ex._max_ 560 nm, em._max_ 585 nm) for the indicated timepoints at 37°C. Dyes were removed and cells were washed with PBS, which was replaced with optimem at 37°C. Where experiments were carried out using the ion channel inhibitor TEA (10 mM, or a no-drug control), cells were pre-treated with drug prior to dye addition and then maintained throughout the experiment and imaging. Cells were heated to 37°C and imaged on an inverted 510-META laser-scanning confocal microscope at the indicated timepoints. To assess total fluorescence of AG4 in live cells, the IncuCyte ZOOM imaging system was used to determine mean fluorescence intensity (GCU, green calibration units).

## Supporting information

S1 Fig K^+^ primed virions enter the endocytic system. **(A)** Pre-infection priming of BUNV virions was carried out using pH 6.3 (-K^+^) CTL buffer and pH 6.3 high [K^+^] buffer. Primed virions were added to cells on ice, followed by washing in cold DMEM to remove unbound virions. Warm pH 5.0 fusion buffer (DMEM containing 50 mM sodium citrate) was added to cells for a 2 min pulse at 37°C, alongside control samples incubated with either an acid-bypass control buffer (DMEM, 50 mM HEPES, 20 mM NH_4_Cl, at pH 7.4) or DMEM alone. Cells were warmed and acid-bypass control buffer was added to all except the DMEM control wells, where DMEM was added to allow endocytic entry of viruses, for confirmation of virus priming. Infected cells were incubated for 17 hrs and lysed and BUNV-N assessed by westen blot (n = 3). **(B)** Cells were treated 30 min prior to infection with media ± NH_4_Cl and then infected with pH 6.3 primed virions (± KCl) in the presence or absence of NH_4_Cl throughout infection. Cells were lysed 18 hpi and BUNV-N assessed as in **(A)** (n = 3).
(TIF)

S2 Fig Verification of SYTO82/DiDvbt BUNV. **(A)** Plaque assay of dual labelled BUNV fractions showing infectivity is not compromised following fluorescent labeling. **(B) (i-ii)** Example images of infected A549 cells confirming the complete overlap of SYTO82-DiDvbt signals assessed by line scan analysis (Zen software). Images were taken 8 hrs post-infection and are representative of ≥ 200 cells. Scale bar = 10 μM. **(C)** Infection of HAP-1 cells with dual labelled BUNV as in (**B)**. Images were taken 8 hrs post-infection and are representative of ≥ 30 cells.
(TIF)

S3 Fig AG4 distribution is unaffected by the time of labelling. AG4 (10 μM) was added to A549 cells for the indicated timepoints to allow endosomal uptake, alongside **(A)** 488-labelled EGF or **(B)** Magic Red cathepsin B dye. Dyes were subsequently removed and live cells were imaged as in Fig 3. Representative images are shown (n≥40 cells). Scale bar = 10 μM.
(TIF)

S4 Fig Confirmation of movement of BUNV into late endosomes. **(A)** Example image of infected A549 cells confirming the overlap of SYTO82-DiDvbt-EGF signals assessed by line scan analysis (Zen software). Images were taken 4 hrs post-infection and are representative of ≥ 100 cells. **(B)** As in **(A)** assessing overlap of SYTO82-DiDvbt in cells transfected with Rab7 GFP. Scale bar = 10 μM.
(TIF)

S5 Fig BUNV moves into cells with EGF and Tf and traffics to endosomes that lack Tf at later timepoints. **(A)** Cells were infected with SYTO82/DiD-BUNV for 1 hr at 4°C, then heated to 37°C and infection was allowed to proceed for 20 mins in the presence of biotinylated EGF-488. Confocal images were taken at t = 20 mins and representative live images of BUNV-EGF-488 fluorescence taken at 20 second intervals are shown. **(B**) Cells were infected as in **(A)** in the presence of 488-labelled Tf and imaged at the indicated timepoints. Images are representative of ≥40 cells. Scale bar = 10 μM.
(TIF)

S6 Fig Proposed model of BUNV K^+^ dependence. **(A)** BUNV enters cells and is internalised into EEs and trafficked to LEs. [K^+^] increases down the endocytic pathway expedited by K^+^ channels on endosomal membranes, peaking in late endosomes. This increase, coupled to decreasing pH, establishes an environment that facilitates BUNV endosomal escape. **(B)** In cells treated with the K^+^ channel inhibitor TEA, endosomal K^+^ channels are blocked. The [K^+^] increase down the endocytic pathway is inhibited. This results in the accumulation of K^+^ in the more acidic environment of lysosomes. Under these conditions, BUNV is unable to meet the pH/K^+^ environment required for endosomal escape. BUNV virions are therefore arrested within the endocytic network (in lysosomes) under low pH conditions that cause the BUNV virions to be irreversibly non-infectious.
(TIF)
