# Supplementary figures and images for: Bunyavirus requirement for endosomal K+ reveals new roles of cellular ion channels during infection
Source: PLoS Pathog. 2018 Jan 19;14(1):e1006845. doi: 10.1371/journal.ppat.1006845 (PMC5805358; doi:10.1371/journal.ppat.1006845)

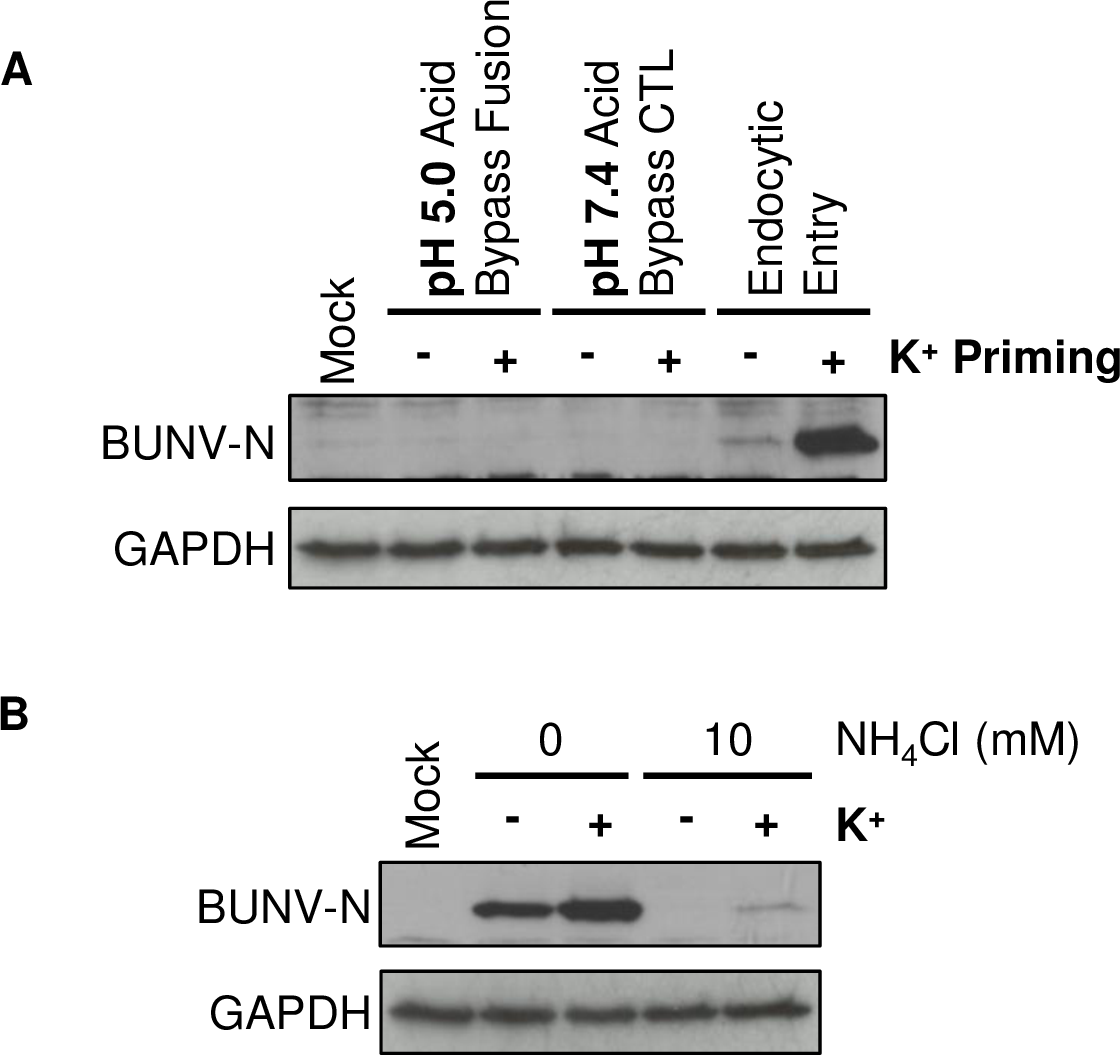

Supplement: S1 Fig — (A) Pre-infection priming of BUNV virions was carried out using pH 6.3 (-K+) CTL buffer and pH 6.3 high [K+] buffer. Primed virions were added to cells on ice, followed by washing in cold DMEM to remove unbound virions. Warm pH 5.0 fusion buffer (DMEM containing 50 mM sodium citrate) was added to cells for a 2 min pulse at 37°C, alongside control samples incubated with either an acid-bypass control buffer (DMEM, 50 mM HEPES, 20 mM NH4Cl, at pH 7.4) or DMEM alone. Cells were warmed and acid-bypass control buffer was added to all except the DMEM control wells, where DMEM was added to allow endocytic entry of viruses, for confirmation of virus priming. Infected cells were incubated for 17 hrs and lysed and BUNV-N assessed by westen blot (n = 3). (B) Cells were treated 30 min prior to infection with media ± NH4Cl and then infected with pH 6.3 primed virions (± KCl) in the presence or absence of NH4Cl throughout infection. Cells were lysed 18 hpi and BUNV-N assessed as in (A) (n = 3). (TIF) [file ppat.1006845.s001.tif]

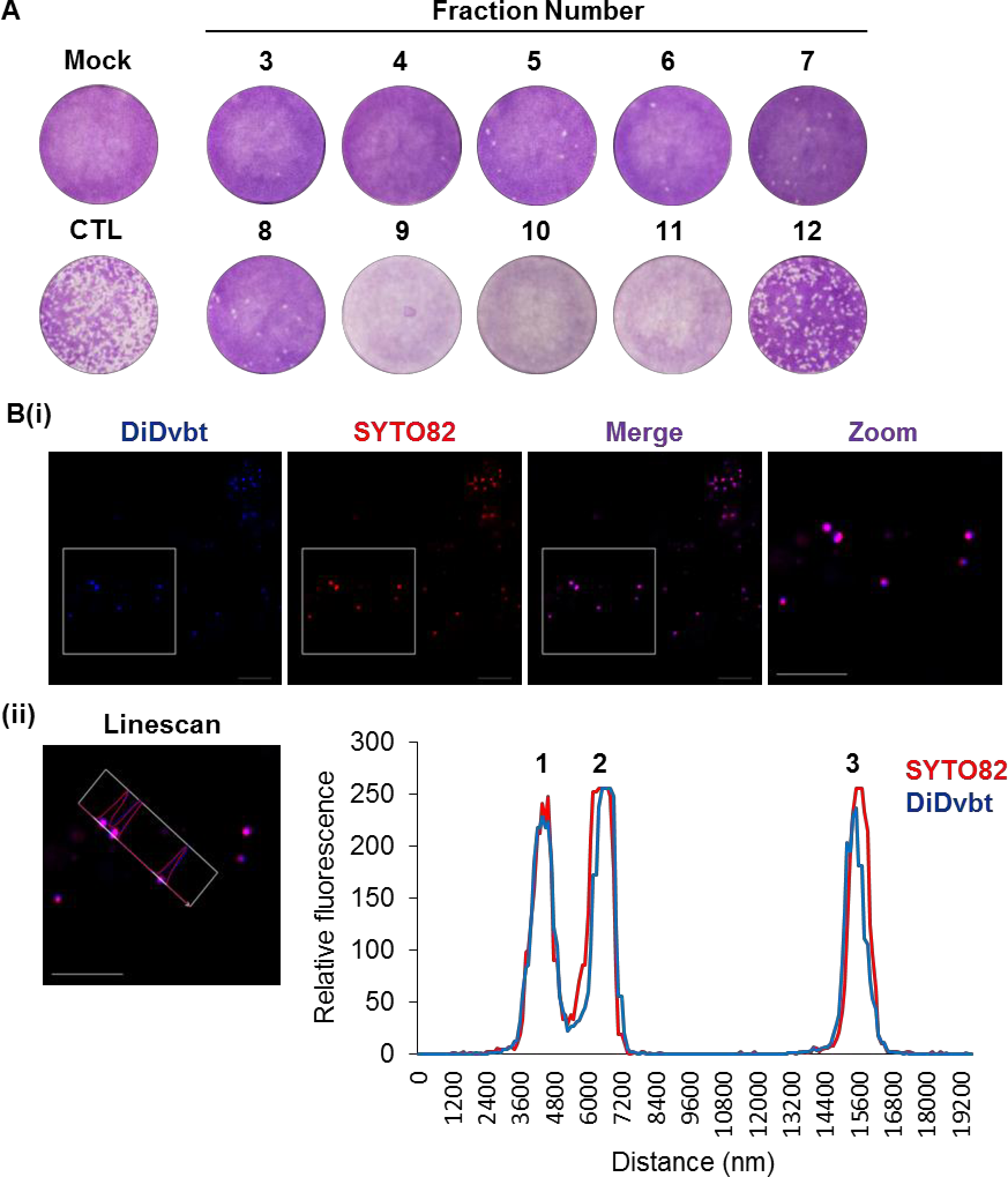

Supplement: S2 Fig — (A) Plaque assay of dual labelled BUNV fractions showing infectivity is not compromised following fluorescent labeling. (B) (i-ii) Example images of infected A549 cells confirming the complete overlap of SYTO82-DiDvbt signals assessed by line scan analysis (Zen software). Images were taken 8 hrs post-infection and are representative of ≥ 200 cells. Scale bar = 10 μM. (C) Infection of HAP-1 cells with dual labelled BUNV as in (B). Images were taken 8 hrs post-infection and are representative of ≥ 30 cells. (TIF) [file ppat.1006845.s002.tif]

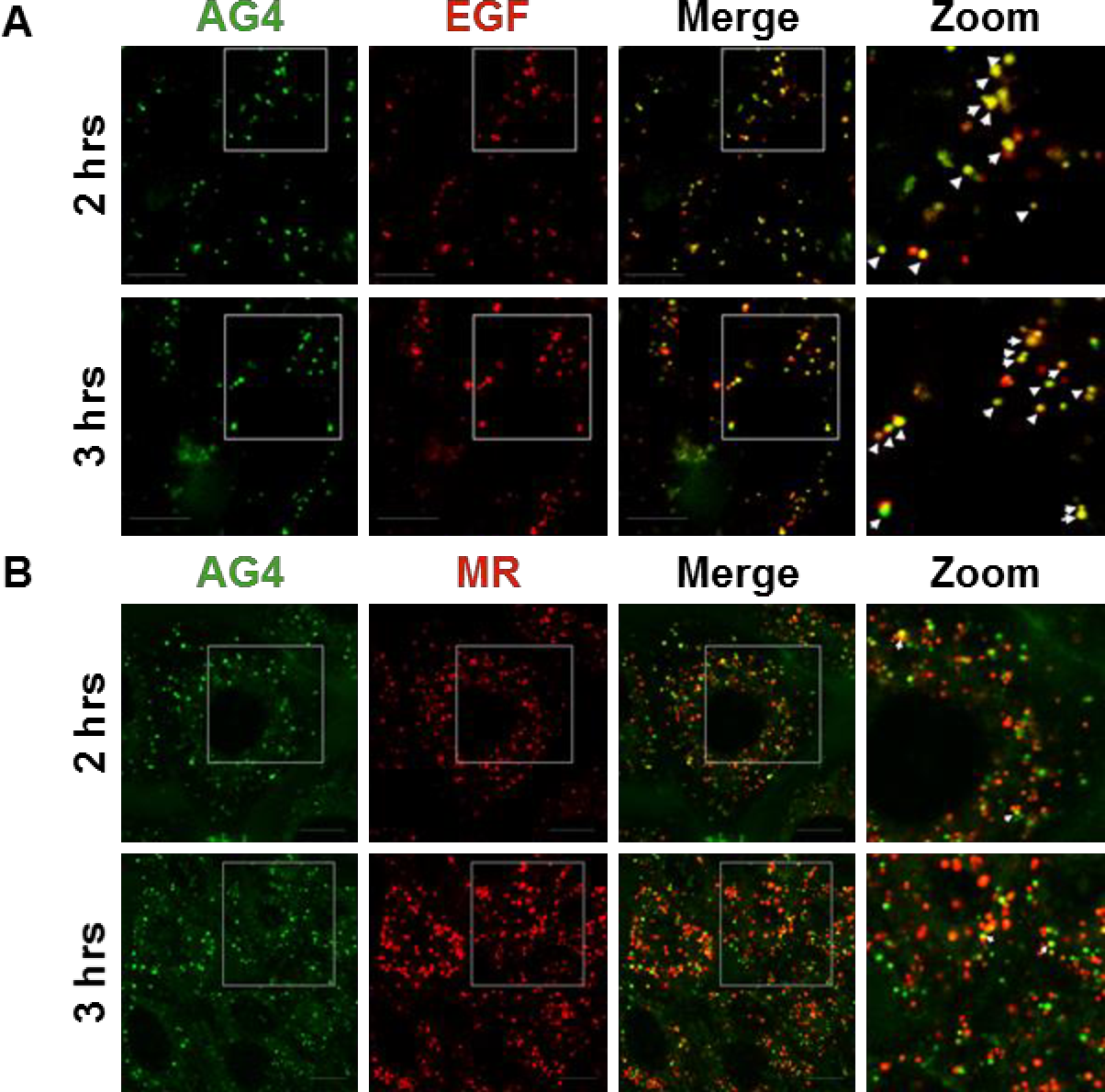

Supplement: S3 Fig — AG4 (10 μM) was added to A549 cells for the indicated timepoints to allow endosomal uptake, alongside (A) 488-labelled EGF or (B) Magic Red cathepsin B dye. Dyes were subsequently removed and live cells were imaged as in Fig 3. Representative images are shown (n≥40 cells). Scale bar = 10 μM. (TIF) [file ppat.1006845.s003.tif]

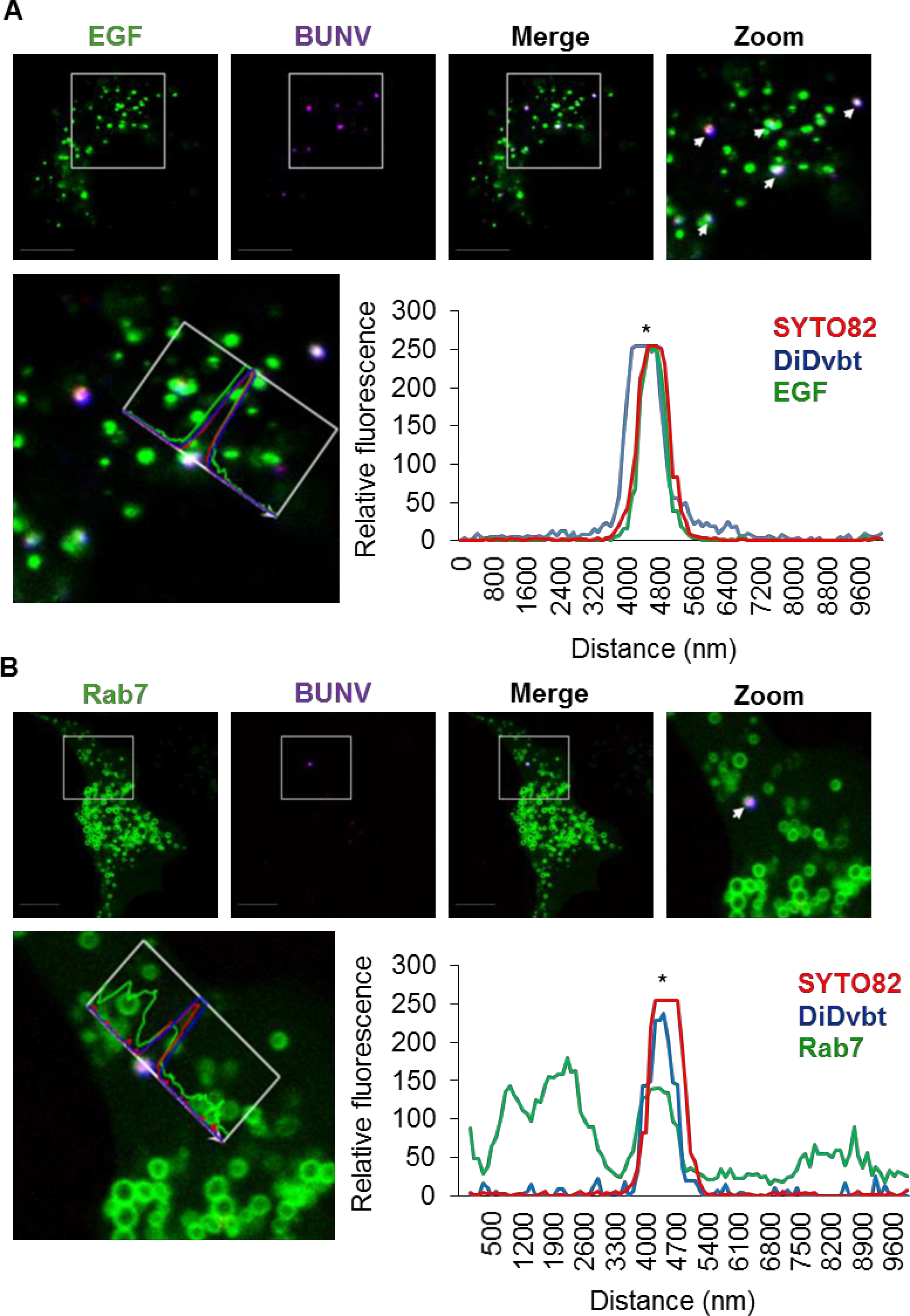

Supplement: S4 Fig — (A) Example image of infected A549 cells confirming the overlap of SYTO82-DiDvbt-EGF signals assessed by line scan analysis (Zen software). Images were taken 4 hrs post-infection and are representative of ≥ 100 cells. (B) As in (A) assessing overlap of SYTO82-DiDvbt in cells transfected with Rab7 GFP. Scale bar = 10 μM. (TIF) [file ppat.1006845.s004.tif]

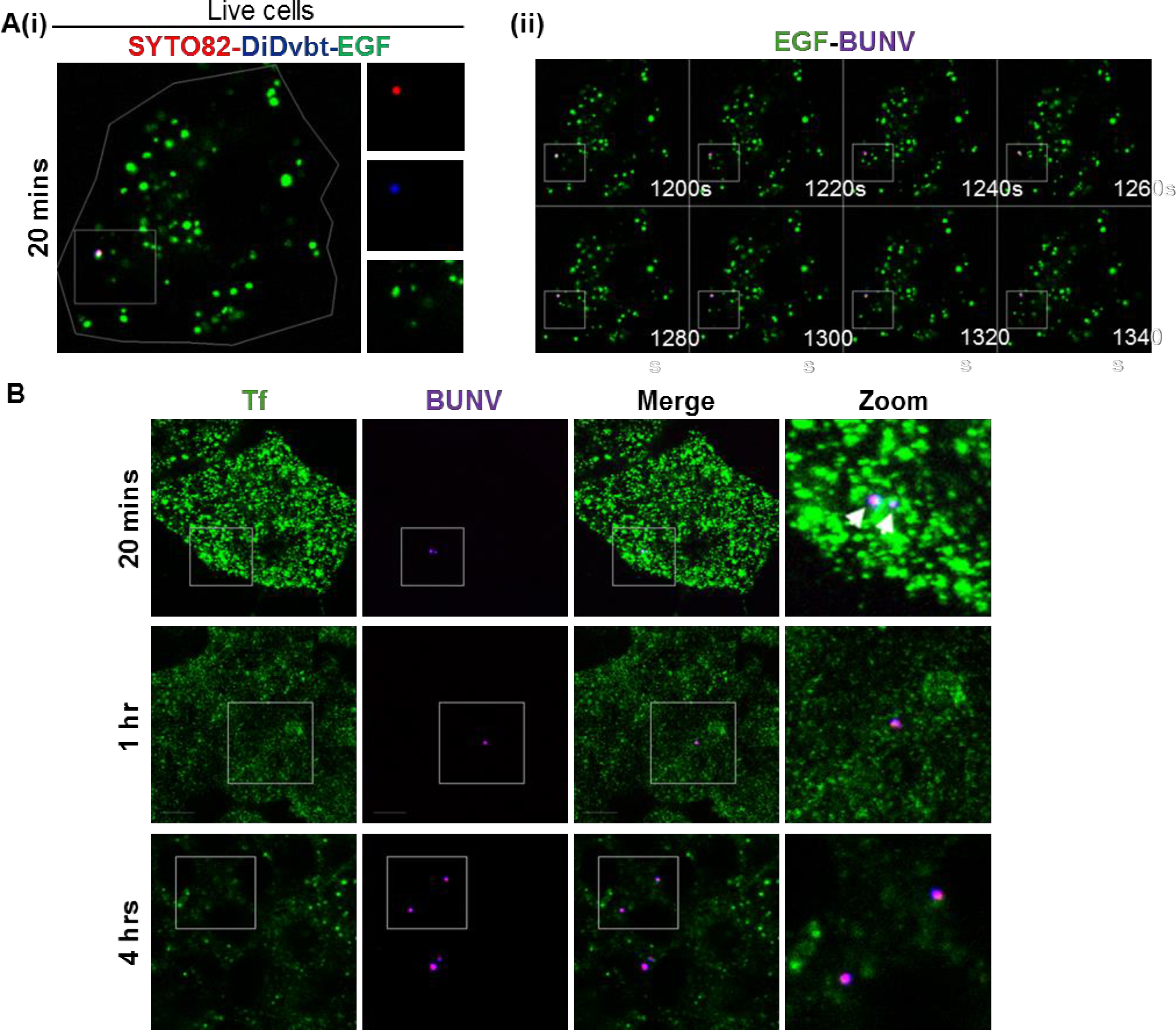

Supplement: S5 Fig — (A) Cells were infected with SYTO82/DiD-BUNV for 1 hr at 4°C, then heated to 37°C and infection was allowed to proceed for 20 mins in the presence of biotinylated EGF-488. Confocal images were taken at t = 20 mins and representative live images of BUNV-EGF-488 fluorescence taken at 20 second intervals are shown. (B) Cells were infected as in (A) in the presence of 488-labelled Tf and imaged at the indicated timepoints. Images are representative of ≥40 cells. Scale bar = 10 μM. (TIF) [file ppat.1006845.s005.tif]

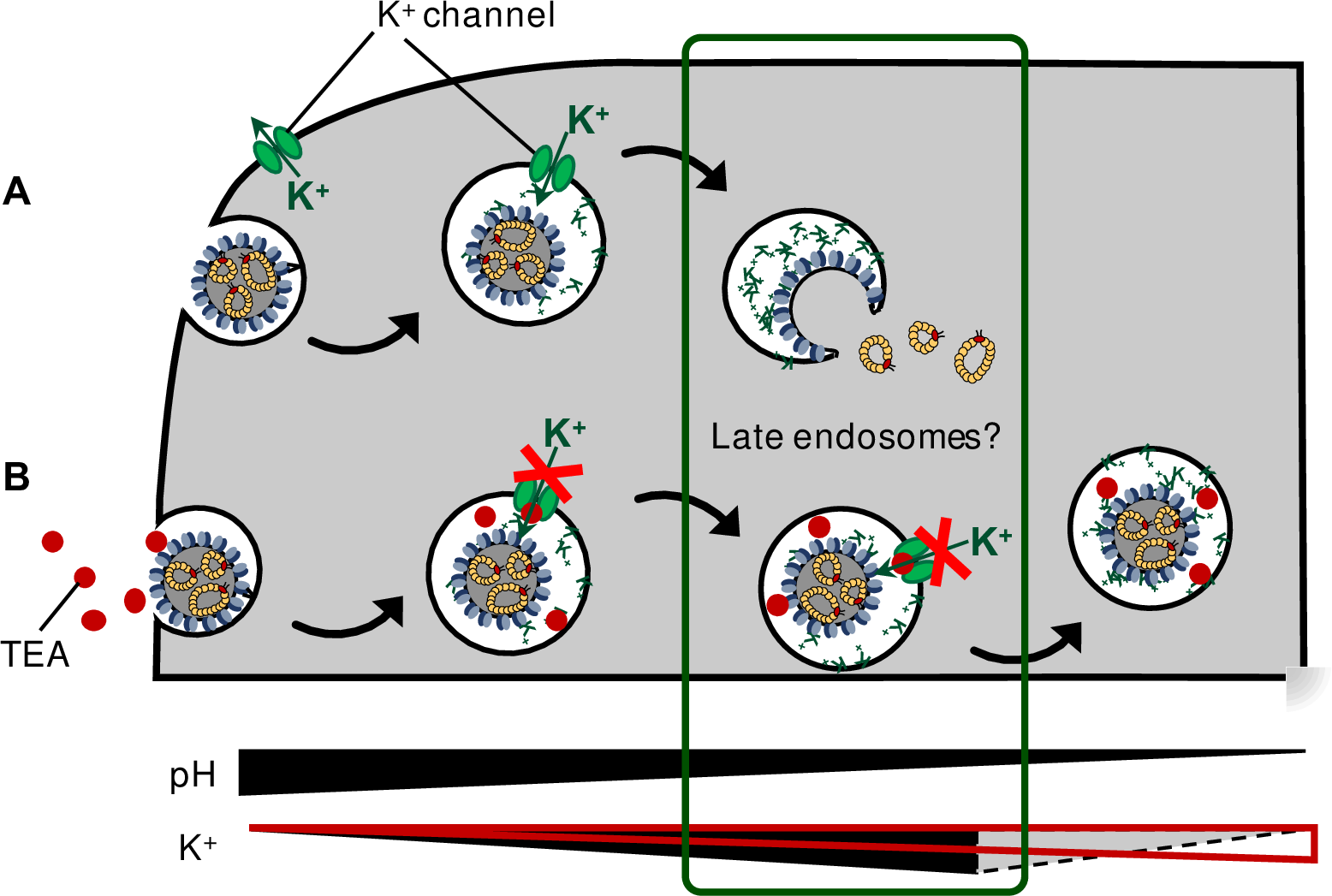

Supplement: S6 Fig — (A) BUNV enters cells and is internalised into EEs and trafficked to LEs. [K+] increases down the endocytic pathway expedited by K+ channels on endosomal membranes, peaking in late endosomes. This increase, coupled to decreasing pH, establishes an environment that facilitates BUNV endosomal escape. (B) In cells treated with the K+ channel inhibitor TEA, endosomal K+ channels are blocked. The [K+] increase down the endocytic pathway is inhibited. This results in the accumulation of K+ in the more acidic environment of lysosomes. Under these conditions, BUNV is unable to meet the pH/K+ environment required for endosomal escape. BUNV virions are therefore arrested within the endocytic network (in lysosomes) under low pH conditions that cause the BUNV virions to be irreversibly non-infectious. (TIF) [file ppat.1006845.s006.tif]
